# Interactions Involving Glycine and Other Amino Acid Neurotransmitters: Focus on Transporter-Mediated Regulation of Release and Glycine–Glutamate Crosstalk

**DOI:** 10.3390/biomedicines12071518

**Published:** 2024-07-08

**Authors:** Luca Raiteri

**Affiliations:** 1 Pharmacology and Toxicology Section, Department of Pharmacy (DIFAR), University of Genoa, 16148 Genoa, Italy; luca.raiteri@unige.it; 2 Inter-University Center for the Promotion of the 3Rs Principles in Teaching & Research (Centro 3R), 16148 Genoa, Italy

**Keywords:** glycine, glutamate, GABA, glycine transporter 1 (GlyT1), glycine transporter 2 (GlyT2), NMDA receptors, neurotransmitter release

## Abstract

Glycine plays a pivotal role in the Central Nervous System (CNS), being a major inhibitory neurotransmitter as well as a co-agonist of Glutamate at excitatory NMDA receptors. Interactions involving Glycine and other neurotransmitters are the subject of different studies. Functional interactions among neurotransmitters include the modulation of release through release-regulating receptors but also through transporter-mediated mechanisms. Many transporter-mediated interactions involve the amino acid transmitters Glycine, Glutamate, and GABA. Different studies published during the last two decades investigated a number of transporter-mediated interactions in depth involving amino acid transmitters at the nerve terminal level in different CNS areas, providing details of mechanisms involved and suggesting pathophysiological significances. Here, this evidence is reviewed also considering additional recent information available in the literature, with a special (but not exclusive) focus on glycinergic neurotransmission and Glycine–Glutamate interactions. Some possible pharmacological implications, although partly speculative, are also discussed. Dysregulations in glycinergic and glutamatergic transmission are involved in relevant CNS pathologies. Pharmacological interventions on glycinergic targets (including receptors and transporters) are under study to develop novel therapies against serious CNS pathological states including pain, schizophrenia, epilepsy, and neurodegenerative diseases. Although with limitations, it is hoped to possibly contribute to a better understanding of the complex interactions between glycine-mediated neurotransmission and other major amino acid transmitters, also in view of the current interest in potential drugs acting on “glycinergic” targets.

## 1. Introduction

### 1.1. Glycine and Its Interactions with Other Neurotransmitters

Glutamate (Glu), GABA, and Glycine (Gly) are the three most known amino acid neurotransmitters (NTs) in the Central Nervous System (CNS). While Glu and GABA are, respectively, the major excitatory and inhibitory NTs in many CNS areas, Gly exerts a dual role in neurotransmission: it is usually inhibitory when it activates the strychnine-sensitive ionotropic Gly receptors (GlyRs) [1,2] but it also plays pivotal roles in excitatory neurotransmission as a co-agonist of Glu at the NMDA receptor (NMDAR) [3]. Moreover, Gly further participates in excitatory neurotransmission through “excitatory Gly receptors” of NMDA-type composed of GluN1 and GluN3A subunits [4,5,6,7], activated by Gly alone. Very recently, novel neuromodulatory actions of Gly have been reported, mediated by newly discovered “metabotropic” Gly receptors [8].

Similarly to many other aspects of neurotransmission, interactions involving Gly and other NTs are not completely clarified. Functional interactions between two different NTs include the regulation of NT release by another transmitter/modulator, usually through the activation of receptors expressed on the releasing neurons. Release-regulating receptors can often be located on axon terminals of the releasing neurons as “presynaptic receptors” [9,10]. However, as recalled in Section 1.2, other non-receptor mediated mechanisms have been established, through which regulation of NT release can occur.

Many functional interactions involving Gly and other NTs are known. Ionotropic GlyRs participate in inhibitory neurotransmission, especially in the spinal cord and brainstem [2]. Through GlyRs, Gly regulates the release of different NTs including Glu and GABA, among others (see, for instance, [11,12,13]; see [2] for a review). Glutamatergic NMDARs control the release of many other transmitters [14], therefore Gly is also involved in such modulations because it is a co-agonist of Glu at NMDAR. Conversely, several transmitters/modulators can regulate the release of Gly. For instance, Gly release is regulated by presynaptic ionotropic and metabotropic Glu receptors [15,16], serotonin 5-HT1B/D receptors [17] in the spinal cord, and presynaptic nicotinic receptors in rat hippocampus [18].

Crosstalk between Gly and Glu is of particular physiological and pathological interest [19] and Gly–Glu interactions have pharmacological implications because Gly is also a co-agonist of glutamatergic NMDARs [19,20,21]. Interactions between Gly and GABA are also of particular relevance for several reasons, including that the two amino acids can behave as inhibitory cotransmitters in certain CNS areas (the spinal cord and cerebellum) [22,23,24,25,26,27,28,29].

As introduced above, besides receptor-mediated regulation, other less known possible mechanisms of reciprocal modulation of NT release have been studied; these mechanisms, which are transporter-mediated and involve, in particular, the amino acid NTs Gly, GABA, and Glu, are briefly recalled in Section 1.2.

### 1.2. Functional Interactions Involving Gly and Other Major Amino Acid NTs: A Focus on Transporter-Mediated Mechanisms

**Figure 1:**
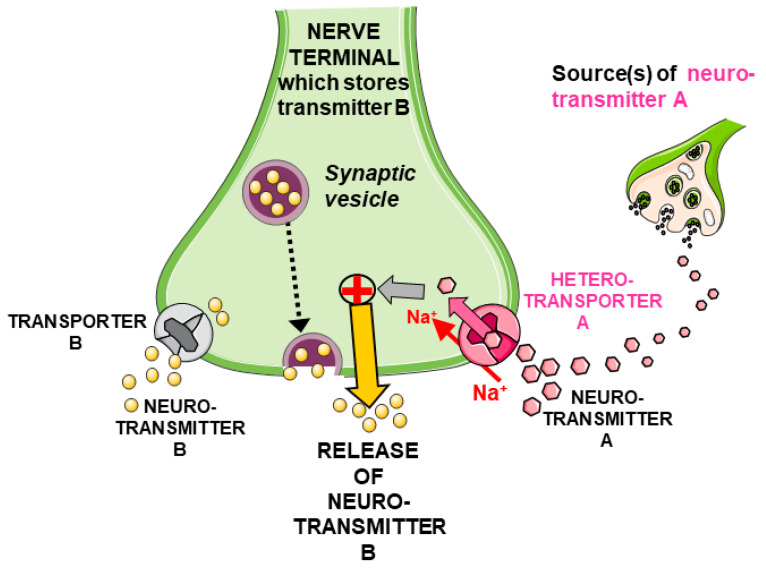
Representative scheme of the stimulation of release of neurotransmitter “B” following activation of the “heterotransporter A” by “neurotransmitter A”. This release can occur through different mechanisms, mostly non-exocytotic in the case of amino acid NTs, as indicated by the evidence discussed in the present work. The whole process is proposed to occur mainly in basal conditions as a mode of neuromodulation that is additional but not alternative to the depolarization-induced “classical exocytosis” of “neurotransmitter B” that depends on action potentials [31].

It is proposed that the release of one NT from nerve terminals can be regulated by another transmitter, also through transporter-mediated mechanisms (for reviews, see [30,31,32]). Briefly (see Figure 1), a given “neurotransmitter A” could regulate the release of “neurotransmitter B” through mechanisms triggered by the activation of “A-selective” Na^+^-dependent transporters able to mediate the uptake of A in the same nerve terminal, which stores and releases B: the result is the increased release of the latter NT “B”. Transporters selective for the “foreign” transmitter A have been termed as “heterotransporters” [30,31,32]. The foreign transmitter could originate from neighboring neurons and/or glial cells but also from more distant cellular sources, thus mediating, in the latter case, a type of non-synaptic communication [33,34].

Of note, the characteristics of the method originally used to study this phenomenon (superfusion of synaptosomes; see [14,31,35,36]) permit us to suggest that two NT transporters (one selective for B, the other selective for A) are present together on the same nerve terminal, inasmuch as B release is usually determined by measuring the release of the radiolabelled “transmitter B” previously taken up by synaptosomal particles that are, therefore, endowed with transporter B (see [31] for details; see also Section 2).

This phenomenon has been recalled here and is briefly discussed further in Section 2 just because a number of proposed functional interactions among the amino acid transmitters Gly, Glu, and GABA are, indeed, mediated by “heterotransporters”, although this phenomenon is not limited to amino acid NTs [31] (pp. 290–291).

In more recent studies, several transporter-mediated interactions among amino acid NTs were investigated more in detail, as described in the following sections.

### 1.3. Aims of the Study

The present work is intended to review and discuss evidence from studies published during the last two decades (also referring, for completeness, to a number of previous studies) in which heterotransporter-mediated interactions involving Gly, Glu, and GABA were characterized in depth at the nerve terminal level in different CNS areas, adding knowledge of intracellular mechanisms that mediate such interactions and suggesting possible pathophysiological significances, with a special focus on the involvement of glycinergic transmission and Gly–Glu interactions. This evidence is, in part, also reviewed in light of additional recent knowledge. Some possible pharmacological implications are also discussed. Although with obvious limitations that also are addressed, it is hoped to contribute to a better understanding of the many aspects of Gly-mediated neurotransmission and functional interactions with other amino acid NTs, also in view of the current interest in compounds interacting with glycinergic targets (for instance, Gly transporters) as potential novel drugs against neurological and psychiatric disorders (see Section 6 and references therein). To the best of my knowledge, the study series, in the way it is reviewed here, has not been the subject of previous reviews.

## 2. Transporter-Mediated Interactions between Two (Not Only Amino Acid) NTs: Some Considerations

The first evidence of heterotransporter systems appeared almost four decades ago and increased significantly over the years (see [30,31] (pp. 290–291) and references therein, [32]). Many of these systems were detected in our laboratory through functional studies exploiting, firstly, the experimental technique of superfused synaptosomes [35] still considered particularly appropriate in studies of presynaptic mechanisms of NT release [14,31,36,37]. A distinction between transporter-mediated effects and receptor-mediated effects has been possible, exploiting functional pharmacology evidence; in particular, it has been possible to propose that the modulation of release of a given “neurotransmitter B” by “neurotransmitter A” (see Figure 1) is transporter-mediated and not receptor-mediated when the effect of “neurotransmitter A” is insensitive to receptor antagonists but prevented by pharmacological blockers of transporters for “neurotransmitter A” [30,31,32].

Other examples were reported by different research groups, exploiting different methods and describing heterotransporter systems not only in nerve terminals but also at different neuronal sites [31,32]. Moreover, other functions (even unknown) were also attributed to such transporter-mediated systems. Finally, heterotransporter systems were also found in sparse studies on the human CNS (see [31] (pp. 290–291) and references therein).

During the last two decades, a number of studies dealing specifically with the heterotransporter-mediated regulation of release among amino acid transmitters were carried out with the aim of extending knowledge of such multiple systems more in-depth, in different experimental conditions. Very often, these studies involved glycinergic neurotransmission, also in relation to the increasing advances in the field. Of note, immunocytochemistry approaches with the same synaptosomal preparations have provided important and complementary information, confirming many functional data obtained in NT release studies [28,38,39,40,41,42].

The presence of transporters for different NTs on the same neuron is, to date, not surprising. With regard to Gly and Glu and interactions between the two NTs, different studies [13,19,43,44,45,46] have described functional, immunohistochemical, and biochemical evidence of similar transporter-mediated effects of Gly on Glu release and of the presence of GlyT1 transporters on glutamatergic nerve terminals (which, according to recent reports [47,48,49], are often endowed with glutamatergic Excitatory Amino Acid Transporters 2, EAAT-2). Moreover, in general, coexistence on the same neuron of two (or more) transporters for different NTs can be associated with cotransmission [28,50,51].

As stated before, the main focus of the present work involves glycinergic neurotransmission, although other main amino acid transmitters are also considered. In Section 3, Section 4 and Section 5, different studies of transporter-mediated interactions involving Gly, Glu, and GABA and leading to the reciprocal regulation of release are reviewed in detail.

## 3. Transporter-Mediated Interactions between Gly and Glu

Interactions between Gly and Glu are of particular relevance because of two main reasons: (i) their role as co-agonists at NMDARs and (ii) their role in the excitation/inhibition balance. Transporter-mediated interactions between the two amino acids at the nerve terminal level, leading to the reciprocal regulation of release, were first described in the rodent cerebral cortex and spinal cord [52]. Later, reciprocal Gly–Glu interactions mediated by their transporter systems have been the subject of different studies performed over the years in our laboratory to investigate, in detail, the mechanisms involved as well as possible pathophysiological implications in different CNS areas, as outlined in Section 3.1, Section 3.2, Section 3.3 and Section 3.4.

### 3.1. Gly-Evoked Glu Release from Spinal Cord Nerve Terminals

**Figure 2:**
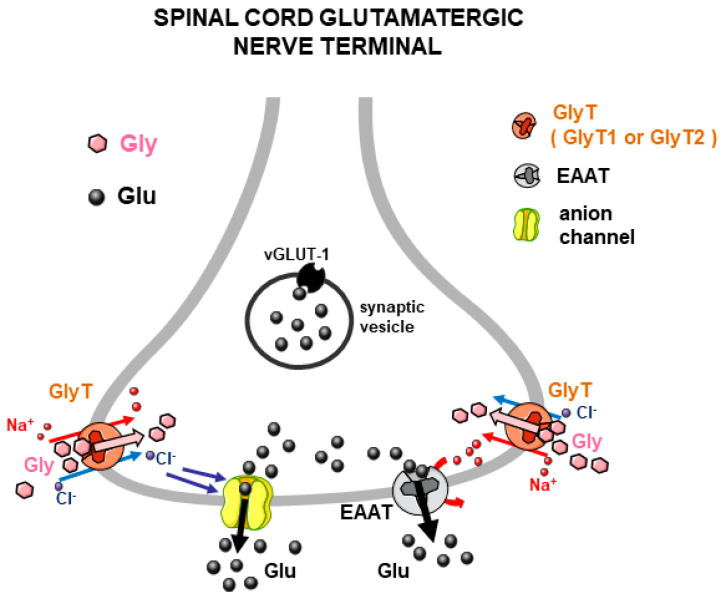
Representation of the Glu release evoked by Gly through activation of heterotransporters (either of GlyT1 or GlyT2 type) from spinal cord glutamatergic, vGLUT-1 positive nerve terminals, according to the evidence described in Section 3.1. Red curved arrow highlights the reversal of transporters for Glu, likely facilitated by Na^+^ entry in nerve terminals due to Gly/Na^+^ cotransport (red straight arrows: Na^+^ ions entry; light blue arrows: Cl^−^ ions entry; black arrows: Glu efflux).

The effect of Gly on the release of Glu from mouse spinal cord nerve terminals was investigated in detail, using purified synaptosomes, to minimize the contribution of astrocyte-derived particles (gliosomes) in the measurement of the NT released [38]. Special attention was given to the study of this functional interaction in the spinal cord because, in parallel investigations, it exhibited pathological interest (see Section 3.2). Similar to results from other studies dealing with the modulation of NT release through heterotransporter systems, the effect of Gly was blocked by selective GlyT1 and GlyT2 transporter blockers while it was insensitive to antagonists of the known receptors activated by Gly [38]. It was established that Gly elicited Glu release through the activation of Glycine transporter 1 (GlyT1)- and Glycine transporter 2 (GlyT2)-type heterotransporters located on spinal cord glutamatergic nerve terminals. It was proposed, therefore, that GlyT1 and GlyT2 transporters, in addition to their preferential localization on astrocytes and glycinergic nerve terminals, respectively, also existed on some spinal Glu-releasing nerve terminals (as “heterotransporters”) and that their activation by Gly triggered internal events (as a consequence of cotransport of Na^+^ and Cl^−^ ions), leading to the stimulation of Glu release by a dual mechanism: (i) exit of Glu through anion channels and (ii) in part, release by reversal of Glu transporters ([38]; see Figure 2). Immunocytochemical evidence confirmed the presence of Gly heterotransporters on vesicular Glu Transporter 1 (vGLUT1)-positive (glutamatergic) nerve terminals in agreement with the functional data. More recent research [42] aimed to understand more details of these functional interactions in an animal model of neurodegenerative disease (as described below, Section 3.2), and further immunocytochemical and functional evidence confirmed the expression of GlyT1 and GlyT2 heterotransporters in mouse spinal cord glutamatergic nerve terminal membranes and their function (enhancement of Glu release).

### 3.2. The Excessive Gly-Evoked Glu Release in an Animal Model of Amyotrophic Lateral Sclerosis

**Figure 3:**
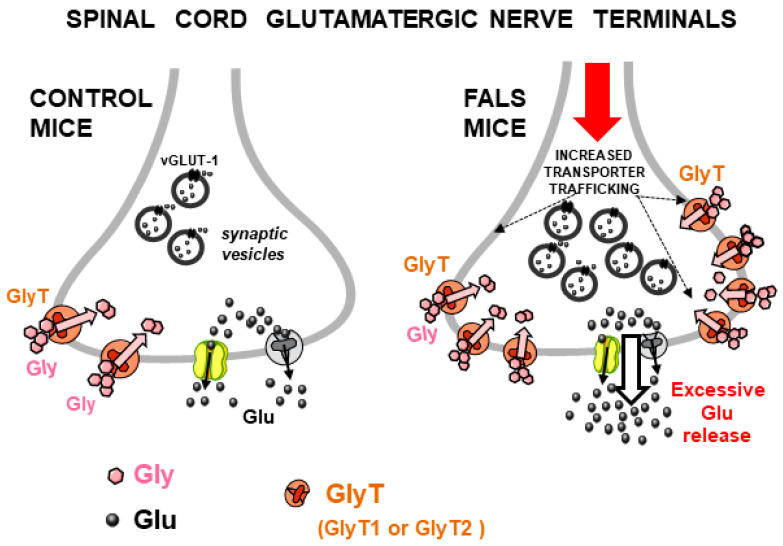
**Left**: Release of Glu induced by Gly through activation of GlyT1/GlyT2 heterotransporters from spinal cord glutamatergic nerve terminals in control mice (**left**) and FALS mice (**right**). **Left panel** is representative of the situation already depicted in Figure 2 (see above) and reported here for comparison. **Right**: the constitutively excessive exocytotic activity in spinal glutamatergic nerve terminals of FALS mice leads to increased trafficking of GlyT1 and/or GlyT2 heterotransporters to the glutamatergic nerve terminal plasma membrane (black dotted arrows) in FALS mice, thus explaining the excessive effect of Gly transporter activation on Glu release (Section 3.2). Arrow with black outline: excessive release of Glu.

A concomitant study [53] investigated the Gly-induced Glu release from mouse spinal cord nerve terminals in a transgenic mouse model of the familial form of amyotrophic lateral sclerosis (FALS), namely SOD1-G93A(+) mice [54], here termed “FALS mice”. The first result was the finding that Glu release induced by Gly from spinal cord synaptosomes of FALS mice was significantly enhanced in comparison with control mice [53] (see Figure 3, right). Gly could enhance the release of both [^3^H]D-Aspartate (used as a marker of Glu) and GABA, an effect mediated by the activation of Gly heterotransporters since it was prevented by a pharmacological blockade of Gly transporters but not by Gly receptor antagonists like strychnine or 5,7-dichlorokynurenate; the effect of Gly on Glu release (but not on GABA release) was strongly enhanced in the spinal cord of FALS mice [53]. In a subsequent study, furthermore, it was confirmed that Gly heterotransporter-induced release of endogenous Glu was also excessive in FALS mice at presymptomatic stages [55]. These studies suggested that the Gly-evoked Glu release might have pathophysiological significance and that, in particular conditions, endogenous Gly might activate its heterotransporters on glutamatergic nerve terminals in the spinal cord, thus inducing excessive Glu release and possibly contributing to excitotoxicity-induced neurodegeneration.

The Gly-induced excessive Glu release in FALS mice was further studied in a more recent report [42] where GlyT1 and GLYT2 expression and function in spinal cord glutamatergic nerve terminal membranes were studied after inducing exocytosis in samples from control and FALS mice, the latter endowed with constitutively excessive efficiency of exocytotic mechanisms, as established previously [56]. Based on immunohistochemical and functional results, it was proposed here that the excessive efficiency of exocytosis in spinal glutamatergic nerve terminals of FALS mice, in addition to excessive Glu release leading to excitotoxicity, also promoted increased trafficking of GlyT1 and GlyT2 heterotransporters to the plasma membrane (exploiting the same enhanced exocytotic pathways), with the subsequent over-expression of functional GLYT1 and GLYT2 heterotransporters able to further contribute to evoke excessive Glu release ([42], Figure 3, right).

### 3.3. Gly–Glu Interactions in Mouse Cerebellum

**Figure 4:**
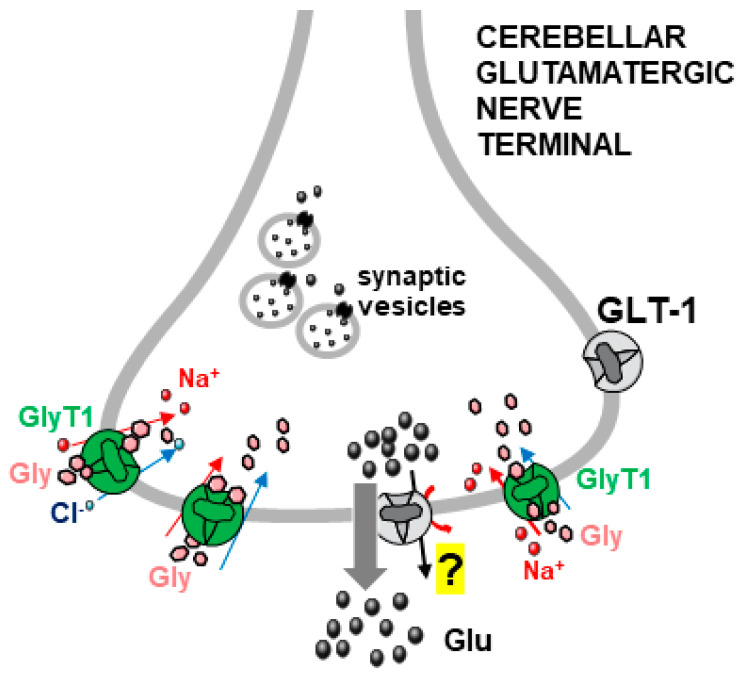
Gly evokes the release of Glu (thick grey arrow) in the cerebellum following activation of GlyT1 transporters likely located on glutamatergic parallel fiber nerve terminals (Section 3.3). Detailed mechanism of Glu release in this CNS area was not investigated (“question mark”), although the similarity with the Gly-evoked Glu release in the spinal cord (Figure 2) suggests that the mechanism involved could be similar and partially involve reversal of the Glu transporter GLT-1/EAAT2 (black thin arrow), found to be colocalized with GlyT1 [28]. Straight red arrows: Na^+^ ions entry; blue arrows: Cl^−^ ions entry; curve red arrow: possible transporter reversal facilitated by Na^+^ ions.

The cerebellum is another CNS area in which transporter-mediated interactions between glycinergic and glutamatergic systems have been studied. According to Cubelos et al. [43], GlyT1 transporters are abundant in the cerebellum, where a significant portion of these transporters is located presynaptically on glutamatergic parallel fibers. We found that Gly also potentiated the basal release of Glu in mouse cerebellum [28]. Also, this effect of Gly was mediated by transporter activation, being mainly counteracted by N-[(3R)-3-([1,10-biphenyl]-4-yloxy)-3-(4-fluorophenyl)propyl]-N-methylglycine (NFPS), a well-known selective GLYT1 transporter blocker. We, therefore, suggested that cerebellar glutamatergic parallel fibers express Gly transporters of the GLYT1-type (in agreement with Cubelos et al. [43]) together with transporters for Glu and that the activation of GlyT1 transporters stimulates Glu release in the cerebellum ([28], Figure 4). Confocal microscopy experiments supported the functional data, showing the coexistence of GlyT1 and glutamate transporter 1 (GLT-1, or EAAT-2) on synaptosomal particles positive to microtubule-associated protein 2 (MAP-2). Of note, although this point has been long controversial, recent reports also support the existence of EAAT-2 transporters, largely expressed in glia, on glutamatergic nerve terminals [47,48,49]. In addition to the functional interaction between Gly and Glu in the cerebellum, the finding that GlyT1 and GLT-1 (EAAT2) transporters coexisted on some cerebellar nerve terminals suggested the possibility that Gly and Glu might be co-stored and perhaps sometimes co-released [28]. This is also in agreement with observations from other groups, supporting the coexistence of glycinergic and glutamatergic elements within the same nerve terminal or neuron, a concept that is compatible with possible, although not fully established, Gly–Glu cotransmission (see [43,44,46,57,58,59,60,61]; see also Section 3.4).

### 3.4. Gly–Glu Interactions in Mouse Hippocampus

As mentioned above, Gly mediates both inhibitory and excitatory functions. Regarding the latter aspect, mostly related to the role of Gly as an NMDAR co-agonist, the hippocampus is a brain region in which many crucial NMDAR-mediated functions are well-studied. Accordingly, dysregulations of Gly-mediated functions, Gly transporters, and Gly–Glu interactions in this area are taken into account in relation to CNS disorders that include neurotoxicity, brain ischemia, epilepsy, depression, and schizophrenia [19,21,46,62,63].

**Figure 5:**
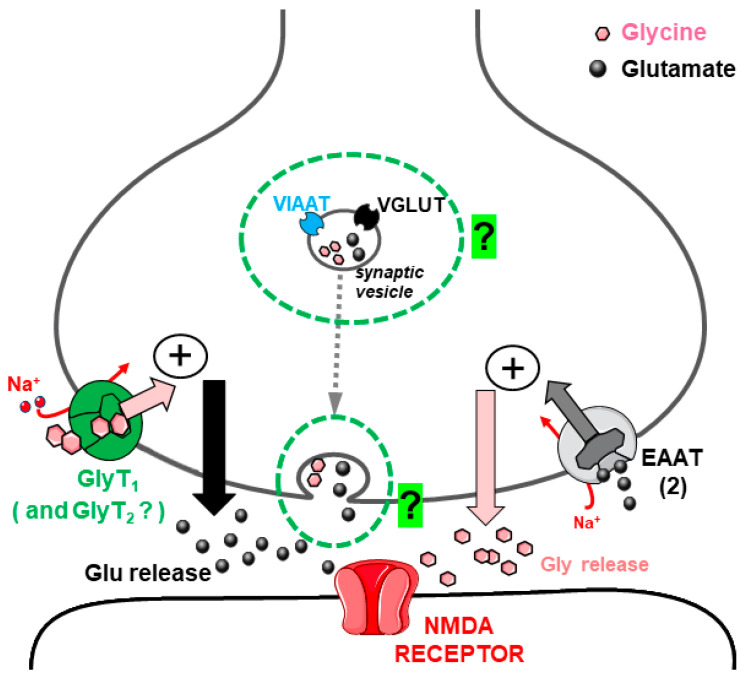
Representation of the transporter-mediated Gly–Glu interactions in a subset of hippocampal nerve terminals in which we proposed that Gly and Glu could be co-stored, on the basis of reported evidence suggesting Gly–Glu cotransmission and of our recent results [64], as described in Section 3.4. The parts circled with a green dashed line are still partly speculative (presence of a question mark); in the presence of cotransmission, Gly and Glu could be released together by exocytosis following depolarization (grey dotted arrow), in agreement with the study by Muller et al. [57], and could also participate in regulating each other’s release (⊕) through activation of their transporters (pink and grey outside-inside arrows). In the absence of cotransmission, the two reciprocal interactions between Gly and Glu, depicted here in the same nerve terminal, would occur in separate (Gly-releasing and Glu-releasing) terminals. The two amino acids can reciprocally regulate their release onto postsynaptic NMDARs (see [64]).

Recently, we extended our studies of interactions between Gly and Glu to the mouse hippocampus [64]. Similarly to the rodent cerebral cortex but, in part, differently from the spinal cord (see [52] and Section 3.1), it was found that both Gly and Glu reciprocally stimulate their release from nerve terminals in the hippocampus through “typical” heterotransporter systems. According to the results, we proposed the coexistence, on a subset of hippocampal nerve terminals, of transporters for Gly and Glu (especially of the EAAT-2 type). Considering that the coexistence on the same nerve terminal of transporters for two different NTs may reflect cotransmission [28], we proposed that our results, together with the above-cited evidence suggesting possible Gly–Glu cotransmission (see Section 3.3 and references therein), could further strengthen such a hypothesis [64]. Although this study did not clearly demonstrate that the two amino acids are cotransmitters, the available information prompted us to propose (in a partially speculative way) that a limited number of mouse hippocampal nerve terminals might co-store Gly and Glu, possibly as cotransmitters, in agreement with the results obtained by Muller et al. [57]. However, regardless of cotransmission, the two amino acids reciprocally regulate each other’s release through their transporters, either in the case of the existence of possibly sparse “mixed” terminals releasing both Gly and Glu or in the case of release from separate (Glu-releasing and Gly-releasing) nerve terminals (see [64]). Thus, Gly-induced Glu release and Glu-evoked Gly release may have physiological relevance, particularly considering their roles as co-agonists of NMDARs; Figure 5 and the related legend describe the hypothesis proposed by the above-mentioned study [64].

## 4. Transporter-Mediated Interactions between GABA and Glu

Although in the present work, the main focus is on interactions involving glycinergic neurotransmission, transporter-mediated interactions between Glu and GABA also deserve attention. These interactions were characterized in depth in the spinal cord (reviewed in [32]). These studies were closely related to others, summarized above (Section 3.1 and Section 3.2). It was, therefore, considered interesting, here, to add more recent information on Glu–GABA interactions.

### 4.1. GABA-Induced Glu Release in the Spinal Cord

**Figure 6:**
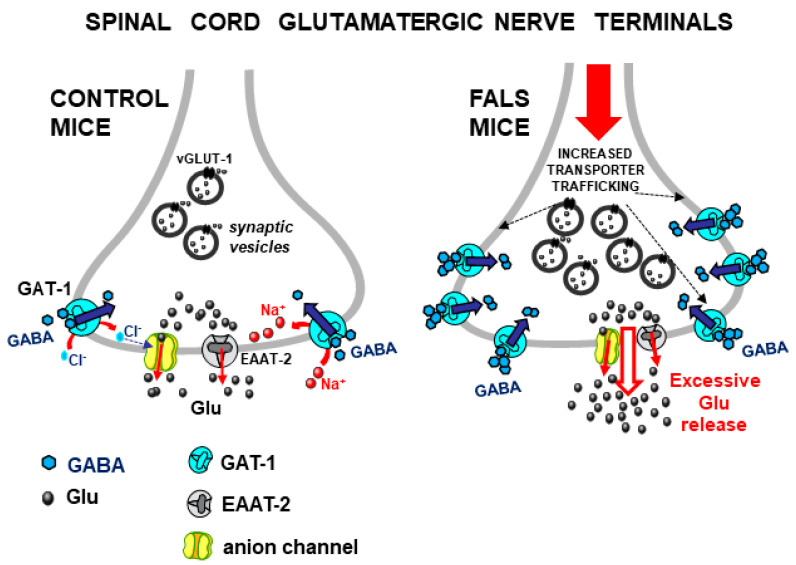
**Left**: Glu release evoked by activation of GAT-1 heterotransporters (blue arrows) from spinal cord glutamatergic, vGLUT-1-positive nerve terminals, according to the evidence proposed (Section 4.1). Curve arrows: Cl^−^ and Na^+^ ions entry. Straight red arrows: efflux of Glu. **Right**: Excessive Glu release (inside-out red straight arrows) evoked by activation of GAT-1 heterotransporters whose “trafficking” to the plasma membranes of glutamatergic nerve terminals of FALS mice (black dotted arrows) is augmented [42] (Section 4.1).

GABA was found able to enhance the release of Glu (and vice versa) through the activation of heterotransporter systems (see [31], pp. 290–291 and references therein). In the mouse spinal cord, the activation of GABA transporters 1 (GAT-1) on vGLUT-1-positive glutamatergic nerve terminals elicited the release of Glu. This release was mediated by the opening of anion channels and, in part, by the reversal of Glu transporters of the EAAT-2 type colocalized on the same nerve terminal [32,39] (see Figure 6, left). Of note, Glu was released through mechanisms that were similar to the mode of exit of Glu following the activation of GlyT1/GlyT2 transporters in the same CNS area (Section 3.1). Similarly to the Gly-evoked Glu release, the GABA-evoked Glu release was also strongly and precociously enhanced in FALS mice [32,55,65] (Figure 6, right).

Later, it was also found that in spinal cord astrocyte-derived gliosomes able to release Glu, the release of [^3^H]D-Aspartate (used as a marker of Glu) was enhanced by the activation of GAT-1 transporters [66], and this enhancement of Glu release from gliosomes was also excessive and precocious in the spinal cords of FALS mice [66].

Finally, the excessive Glu release induced by GABA from spinal cord nerve terminals in FALS mice is linked to the enhanced trafficking of GAT-1 heterotransporters in glutamatergic synapses [42] (Figure 6, right).

Thus, these GABA–Glu interactions also exhibit possible pathophysiological interest. As in the case of Gly transporters (see above), the activation of GAT-1 heterotransporters by GABA triggers stimulatory action on glutamatergic transmission, an effect that is uncommon with respect to most well-known, typically inhibitory, GABA-mediated effects.

### 4.2. Glu–GABA Interactions in the Spinal Cord

Finally, during a study aimed to explore the relations between the coexistence of NT transporters on the same nerve terminal and possible cotransmission, a set of functional experiments with pharmacological tools that had been previously unavailable, together with novel immunocytochemistry data, confirmed that Glu increases the basal release of GABA by activating Glu transporters located on GABA-releasing nerve terminals, purified from the mouse spinal cord to minimize contamination with astrocyte-derived particles [28]. Confocal microscopy analysis showed the expression and very significant colocalization of GABAergic GAT-1 and glutamatergic GLT-1 (EAAT2) transporters in MAP-2-positive synaptosomal particles, in line with functional data.

Given that Gly and GABA are cotransmitters in the spinal cord, one possible aspect related to these effects, according to data obtained by Ishibashi et al. [26], was the idea that Glu acts in the spinal cord via its heterotransporters as a “modulator of Gly-GABA cotransmission” (see [28]).

## 5. Transporter-Mediated Interactions between Gly and GABA

**Figure 7:**
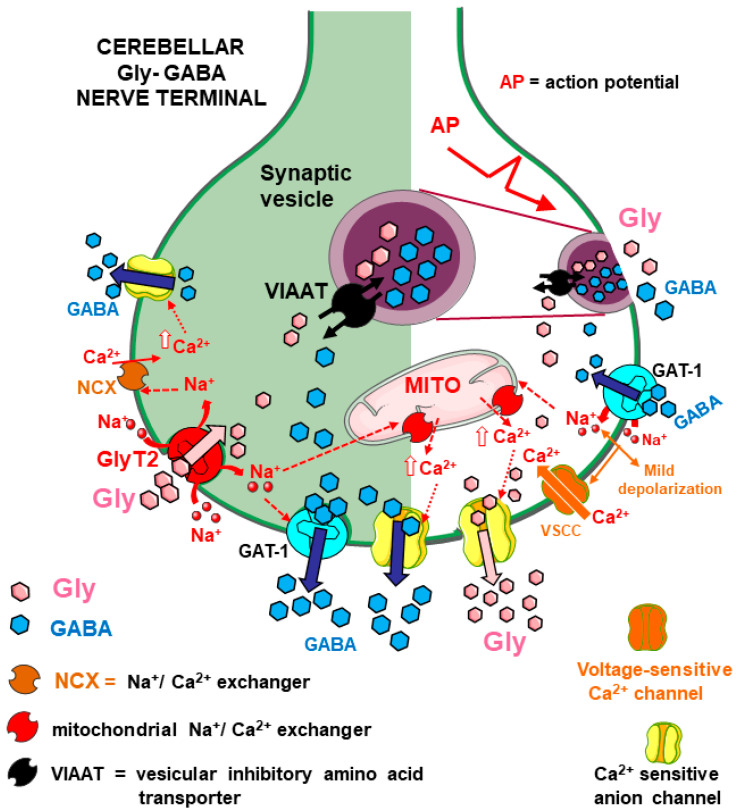
Representation of the functional reciprocal interactions between Gly and GABA in the cerebellum, according to the evidence from functional and immunocytochemical data, in nerve terminals where the two amino acids have been proposed to possibly behave as cotransmitters. The left part of the Figure mainly depicts the Gly-evoked GABA release following activation of GlyT2 (pink arrow), where it is proposed that Na^+^ cotransported with Gly (red curve arrows) contributes to GAT-1 reversal and the increase in local internal Ca^2+^ availability through plasmalemmal Na^+^/Ca^2+^ exchangers (NCX) and mitochondrial Na^+^/Ca^2+^ exchangers, finally facilitating the activation of “Ca^2+^-sensitive anion channels”. GABA is released both by GAT-1 reversal and through anion channels [41] (blue arrows). On the other hand (right part of the figure), GABA can induce Gly release following cotransport of Na^+^ through GAT-1 (red curve arrow); it is proposed that Na^+^ leads to increased availability of internal Ca^2+^ through exchange via mitochondrial Na^+^/Ca^2+^ exchangers (red long-dashed arrows) and, in part, by inducing mild depolarization, sufficient to cause the opening of voltage-sensitive Ca^2+^ channels (orange arrows). This leads to entry of Ca^2+^ ions that do not trigger exocytosis but contribute to the activation of “Ca^2+^-sensitive anion channels” (red thin dotted arrow) through which Gly is released (for details, see [69]). Due to the complexity of the scheme, synaptic versus extrasynaptic locations of transporters, channels, and other structures are not reported.

The first evidence of the reciprocal modulation of release between Gly and GABA through the activation of selective transporters proposed to be co-localized on nerve terminals, particularly in the spinal cord and cerebellum, was reported several years ago [67,68]. Later, these interactions were characterized in depth, providing detailed information on the mechanisms involved [40,41,69]. Since large parts of this information are already included in previous reviews [31,70], the topic is here briefly summarized for the purpose of a more complete overview of interactions involving Gly and other amino acid NTs (Section 5.1 and Section 5.2 and Figure 7), and some related discussion will be added (see Section 6.1 and Section 6.2).

### 5.1. Interactions between Gly and GABA in the Spinal Cord

The first detailed information on the mechanisms of these Gly–GABA interactions in the mouse spinal cord suggested that in this area, Gly can stimulate GABA release from nerve terminals through the activation of GlyT1 and GlyT2 transporters located on nerve terminals able to release GABA (and endowed with GABA transporters), while the proposed mechanism of exit of GABA was dual: (i) “non-conventional” exocytosis due to the increase in internal Ca^2+^ availability and, concomitantly, (ii) the reversal of GABA transporters [67]. In a more recent report [40], by exploiting more novel pharmacological tools, we better confirmed the Gly-evoked GABA release through the activation of GLYT1 and GLYT2 transporters from purified mouse spinal cord nerve terminals, while immunocytochemical characterization of the distribution of GLYT1 and GLYT2 on the same spinal cord synaptosomal particles provided important support to functional data, showing the existence of both GLYT1 and GLYT2 transporters on GABAergic nerve terminals (endowed with GAT-1 transporters) [40]. We considered these functional interactions in the spinal cord of particular interest considering the evidence showing that Gly and GABA are inhibitory cotransmitters in the spinal cord [22,25,26,27,29].

### 5.2. Gly and GABA Reciprocally Modulate Their Release in Mouse Cerebellum

More recently, transporter-mediated interactions between Gly and GABA were studied, exploiting functional and immunocytochemical approaches, in mouse cerebellum, another area in which, according to different reports [23,24,71,72,73], the two amino acids can behave as cotransmitters. Gly stimulated GABA release from cerebellar synaptosomes through the activation of GlyT2 transporters, which triggered internal pathways leading to two final events: (i) the release of GABA through GAT-1 transporter reversal and, (ii) quite unusually, the opening of an anion channel sensitive to Ca^2+^ through which the zwitterion form of GABA could be released, as described in detail in [41] (see Figure 7). Conversely, other evidence showed that the activation of GABA transporters of the GAT-1 type stimulated Gly release from cerebellar nerve terminals through multiple pathways that finally led to the release of Gly from cytosol, essentially permitted by the opening of Ca^2+^-sensitive anion channels [69] (see Figure 7).

Overall, these Gly–GABA reciprocal interactions in the cerebellum were proposed to contribute to the regulation of inhibitory transmission in the cerebellum, including “tonic inhibition” [41,69]. The immunocytochemical identification of GlyT2 and GAT1 transporters and their abundant colocalization on the same cerebellar synaptosomal particles [41] supported functional data.

The existence of transporters for different NTs on one nerve terminal and functional interactions between them is compatible with cotransmission [28]. Clearly, these Gly–GABA interactions seem particularly interesting if they occur in cerebellar nerve terminals that store the two amino acids together as cotransmitters (Figure 7).

## 6. Discussion

### 6.1. General Physiological Significance of the Transporter-Mediated Interactions among Amino Acid NTs

#### 6.1.1. Gly, Glu, and GABA as Reciprocal Enhancers of Their Release through Transporter Activation

One of the first proposed physiological functions of heterotransporter-mediated interactions was their role in the modulation of NT release in basal conditions, in addition to the widely known regulation of release through presynaptic receptors, often occurring under depolarization (see [31]).

With regard to Gly, its heterotransporter-mediated enhancing effect on the release of Glu and GABA is mediated by GlyT1 and GlyT2 transporters on nerve terminals of different CNS areas. The final results of these functional interactions could be both excitatory (in the case of Gly-evoked release of Glu) or inhibitory (Gly-evoked release of GABA). Thus, Gly can also contribute to the excitation/inhibition balance via heterotransporter activation. Moreover, in CNS areas where transporter-mediated Gly–Glu interactions occur, these can participate in the regulation of the availability of Gly/Glu at the NMDAR level.

Similarly, the activation of GABAergic GAT1 heterotransporters can also lead to excitatory effects because it positively modulates Glu release (see Section 4), in contrast with the generally inhibitory nature of the GABAergic effects in the CNS that are mediated by GABA receptors.

#### 6.1.2. Transporters Coexistence and Transporter-Mediated Interactions as “Functional Markers” of Cotransmission

The study of reciprocal, transporter-mediated regulation of release between two NTs with the technique of superfused synaptosomes permits us to suggest, from a functional point of view, the coexistence of transporters on the same nerve terminal for the transmitters involved ([31,32]; see Section 1.2 and Section 2). Moreover, in several examples described in this study, immunocytochemical evidence of the colocalization of the transporters under study supports the conclusions from functional data; the coexistence of functional NT transporters on one same nerve terminals can be seen, although not always, as a marker of cotransmission [28].

We recently proposed that in the hippocampus, in agreement with reports that suggest possible Gly–Glu cotransmission in this area, the coexistence of transporters for Gly and Glu on the same nerve terminals and their ability to mediate the reciprocal regulation of release of the two amino acid transmitters can strengthen the hypothesis of Gly–Glu cotransmission in some hippocampal synapses, possibly onto postsynaptic NMDARs (see Section 3.4 and references therein; Figure 5).

In this general view, the evidence briefly reported in Section 5 seems particularly in agreement with different reports indicating that Gly and GABA can behave as cotransmitters in the spinal cord and also in the cerebellum (see Section 5 and references therein).

### 6.2. Transporter-Mediated Interactions among Amino Acid NTs Are Mediated by Multiple Mechanisms

In general, NTs are physiologically released from nerve terminals by exocytosis triggered by action potentials. However, the release of amino acid NTs reciprocally induced via heterotransporter activation occurs mostly through different mechanisms. This is not surprising since this modulation is proposed to occur mainly in basal conditions (i.e., when nerve terminals are not depolarized [31]).

Likely, one initial trigger is the entry of Na^+^ ions cotransported with the transmitter taken up through the heterotransporter (a process that is Na^+^-dependent). Na^+^ influx is electrogenic and can provide some degree of depolarization, as well as a local increase in the Na^+^ concentration in the nerve terminal. This can facilitate the release of a partial amount of the “paired” amino acid NT, available in the cytoplasm, by transporter reversal, as shown in some examples (Section 3.1 and Figure 2; Section 4 and Figure 6; [41] and Figure 7, left part). The permeation of anion channels accounts for a large portion of the Glu released in response to Gly and GABA heterotransporters in the spinal cord (Section 3.1 and Figure 2; Section 4.1 and Figure 6; [42]). The involvement of particular families of anion channels, sensitive to internal Ca^2+^ ions, has been invoked to largely explain the release of the zwitterion form of GABA induced by Gly and, vice versa, the release of Gly induced by GABA in the cerebellum (Section 5.2 and Figure 7). The pathways leading to the opening/activation of anion channels can involve different responses to the initial transporter activation, including changes in the concentrations of Cl^−^, Na^+^, and even Ca^2+^ (see also [70], pp. 9–12).

To conclude, the transporter-mediated interactions among amino acid NTs lead to the modulation of release, which mainly occurs through non-exocytotic mechanisms including release by transporter reversal and through the opening of different types of anion channels.

### 6.3. Possible Pathophysiological Implications of Transporter-Mediated Gly–Glu (and GABA–Glu) Interactions

If the transporter-mediated interactions considered here indeed occur in vivo, their dysregulation may have pathological consequences. The first example, as detailed in Section 3.2 and Section 4.1, is the excessive, precocious Glu release elicited by Gly and GABA heterotransporters in the spinal cord of FALS mice. This possible pathologically relevant phenomenon deserves some comments considering recent developments in research on Gly and GABA transporters by other authors. Additionally, a few other possible examples/hypotheses of pathophysiological implications, although partly speculative, are discussed in Section 6.3.

#### 6.3.1. The Excessive Glu Release Evoked by Gly and GABA in the Spinal Cord in Animal Models of Amyotrophic Lateral Sclerosis

Excessive glutamatergic activity, leading to excitotoxicity and neurodegeneration, likely plays a pivotal role in amyotrophic lateral sclerosis (ALS) [74,75,76,77,78,79,80]. Excessive levels of Glu in the spinal cord can result from different factors, including its excessive and precocious release from neurons and glia [56,75,81,82,83,84,85]. Reliable evidence showed that SOD1-G93A(+) mice exhibit abnormal Glu release due to the excessive efficiency of exocytosis in spinal glutamatergic nerve terminals [56]. As outlined in Section 3.2 and Section 4.1, excessive Glu release triggered by Gly and GABA heterotransporters from Glu-releasing nerve terminals and glial perisynaptic processes in the spinal cord of FALS mice has also been proposed as a possible source of excessive Glu. Moreover, the proposal that abnormal exocytotic mechanisms also lead to augmented trafficking (followed by overexpression) of functional Gly and GABA heterotransporters to the plasma membrane and that this process is responsible for the excessive Glu release triggered by Gly and GABA, suggested that the excessive exocytosis of Glu and the excessive heterotransporter-induced (non-exocytotic) release of Glu are part of the same dysregulation scenario [42] (see Figure 3 and Figure 6), in which excessive Glu release causes or worsens excitotoxicity ([81] (p. 131); [42,86]). Accordingly, the precocious, selective pharmacological inhibition of GlyT1 and/or GlyT2 and/or GAT-1 transporters may provide a dual-beneficial effect: (i) a reduction in Glu release (by blocking heterotransporter-induced Glu efflux) and (ii) enhancement of inhibitory transmission because the uptake of both Gly and GABA would also be inhibited, thus limiting the excitation/inhibition unbalance [42]. Here, some considerations could strengthen the view that such transporter blockers are potentially useful against neurodegenerative disorders in which excitation/inhibition imbalance plays a role: (i) recently increasing evidence seems to further strengthen the idea that the failure of Gly- and/or GABA-mediated inhibitory neurotransmission plays a major role in motor neuron degeneration in ALS [75,76,80]. In this view, potential additional precocious therapies with Gly and/or GABA transporter blockers could indeed make sense, even independently of the occurrence of “heterotransporters”. Furthermore, (ii) recent evidence strengthened the interest in Gly transporter blockers under study as potential drugs against several CNS pathologies [19,21,46] in research on neurodegenerative disorders; in particular, GlyT1 transporter blockers exhibit neuroprotective properties in studies on neurodegenerative diseases [46,62,87]. Although through different mechanisms, these agents counteracted excitotoxicity in animal models of Parkinson’s and Huntington’s disease [88]. Perhaps, a hypothesized therapeutic strategy based on the Gly transporter blockade could be more successful with novel reversible or competitive transporter blockers than with “irreversible” blockers whose side effects could indeed represent relevant drawbacks (see [89]; see also Section 6.3.2).

#### 6.3.2. GlyT1, GlyT2, and GAT-1 Transporters and Pain

One important current field of research involving, among other systems, glycinergic and GABAergic transmission is the search for novel analgesic treatments. Both glutamatergic (excitatory) and glycinergic/GABAergic (inhibitory) neurotransmission play pivotal, opposite roles in pain processing. In particular, to ameliorate the excitation/inhibition imbalance in the dorsal horn of the spinal cord, different authors have proposed strategies to enhance Gly- and/or GABA-mediated inhibitory transmission via the pharmacological inhibition of Gly and/or GABA uptake, exploiting blockers of GlyT1 and/or GlyT2 transporters [89,90,91,92,93,94,95,96] and GABA GAT-1 and GAT-3-type transporters [94,97,98,99,100], although many aspects still need to be clarified. In this view, concomitant use of different drugs can produce additive or synergistic effects. As an example, some authors indicated the possible advantages of concomitant use of both GlyT1 and GlyT2 blockers (see [101,102]). Recently, novel hypotheses have been proposed to explain the possible additional benefits of GlyT1 inhibitors in reducing opioid tolerance [103]. Besides the enhancement of inhibition, an appropriate reduction in excitation could also add some benefits.

Here, again, one could propose that, in the spinal cord, additional possible benefits of the concomitant inhibition of GlyT1, GlyT2, and/or GAT-1 may exist, considering that transporters of GlyT1/GlyT2 and GAT-1 type are also present as “heterotransporters” on subsets of Glu-releasing spinal nerve terminals as discussed in the present work. If this is the case, pharmacological blockers of GlyTs and GAT1 would not only enhance inhibitory transmission but also prevent the heterotransporter-induced release of excitatory Glu, and perhaps help to ameliorate the excitation/inhibition ratio. Again, with regard to Gly transporter blockers, reversible blockers of GlyT1 like bitopertin [92] or similar compounds and the recently developed GlyT2 blockers, often behaving as partial and/or reversible inhibitors [89,104,105,106], may be preferred to avoid the possible serious side effects observed in experimental models with irreversible full transporter blockers [89].

#### 6.3.3. Considerations on Transporter-Mediated Interactions Involving Amino Acid NTs in the Hippocampus

NMDAR hypofunction in specific neuronal circuits is a core feature of schizophrenia (SCZ); for reviews, see [19,21,107]. NMDAR activity can be restored by increasing the levels of Glu co-agonists of NMDARs, namely Gly and/or D-serine, for instance through the inhibition of Gly reuptake with GlyT1 transporter blockers (see [20,107,108] for reviews). Although some disappointing outcomes from clinical trials with the GlyT1 inhibitor bitopertin lowered the original enthusiasm related to GlyT1 inhibitors against SCZ [109,110], novel GlyT1 blockers are still receiving attention in relation to potential benefits against cognitive ([111,112], see also [113] for a review) but also negative [114] symptoms of SCZ and treatment-resistant SCZ (for a review: [115]). Many explanations have been provided for the negative results with bitopertin (see [109,110,116]), including possible counterproductive compensatory effects [19], also considering that beyond GlyT1 and GlyT2, other transport systems can vehiculate Gly [19,117]. Combined additional therapies could be considered to better enhance the impaired NMDARs functions (see [19], p. 9). D-serine is, besides Gly, the main co-agonist of NMDARs, while D-Aspartate (D-Asp) is a very effective NMDAR agonist. These two D-amino acids, as well as drugs able to regulate their endogenous availability at the NMDAR level, are emerging as further potential disease-modifying and augmentation agents in treatment-resistant SCZ ([115,118,119,120,121]; see also [122]).

In a recent work, we described transporter-mediated Gly–Glu interactions in the hippocampus [64] (see Section 3.4). It is here hypothesized that, in such a complex scenario at the synaptic and perisynaptic levels, some of these interactions might be, additionally, taken into account. Regardless of the possible Gly–Glu cotransmission, according to our recent results [64], Gly can contribute to the upregulation of Glu release in the hippocampus by activating GlyT1 transporters, while the activation of EAAT transporters by Glu or other substrates can increase Gly release [64] (Figure 5), possibly also onto NMDARs. If these Gly–Glu interactions occur in vivo, the beneficial effect of inhibitors of the GlyT1-mediated reuptake of Gly (increased availability of Gly at NMDAR) could be partially masked because the GlyT1 blockade also could limit the possible GlyT1-induced Glu release onto NMDAR (see Section 3.1; Figure 5). Perhaps more plausible from a biological point of view is the possible additional benefit of EAAT transporter activation. In addition to the well-known direct agonism in NMDARs, D-Asp is an effective substrate of EAAT transporters, including EAAT “heterotransporters” whose activation enhances the release of Gly in the hippocampus possibly onto NMDARs [64] (see also Figure 5). It may be proposed that an additional benefit of D-Asp could result from its ability to enhance Gly release.

## 7. Limitations

The series of studies here discussed have been performed using isolated nerve terminals (synaptosomes) purified from the CNS areas under study as an experimental model, exploiting functional (NT release experiments) and, often, immunohistochemical methods. However, “heterotransporter” systems have been detected not only on nerve terminals but also at other neuronal sites [34]. More generally, these phenomena have not been recently tested with other models, including neuronal cells in culture and/or slices. Such extensions of these studies are expected to offer additional, possibly complementary information, also exploiting the possible availability of genetically modified mice (see also Section 8).

Concerns can arise from the question of whether the concentrations of NTs able to activate, in vitro, the heterotransporter-mediated functional responses are indeed representative of conditions occurring in vivo. Although it is difficult to precisely know the concentrations of amino acid NTs available, especially in a transient and/or local manner, at nerve terminal levels, on the basis of some reports it can be inferred that the concentrations of substrates able to activate heterotransporters in vitro can have, at least in certain circumstances, pathophysiological significance (see [41] and references therein; [64], p. 10 and p. 12). However, and more generally, limitations also come from the lack of in vivo studies that could significantly strengthen the existing evidence, which, to the best of my knowledge, has not been performed so far due to different issues including the high complexity of the systems involved. To address this challenging task, very useful evidence could be obtained by exploiting microdialysis techniques. Moreover, ongoing advances in different techniques to monitor transmitters in vivo in animal brains, including genetically encoded biosensors together with imaging techniques [123,124], suggest that such methods also could be of help for future developments.

## 8. Concluding Remarks

The main messages from the present work can be summarized as follows: (i) In addition to the widely known functions of Gly, GABA, and Glu in classical neurotransmission that involve the activation of receptors for the three transmitters, it is confirmed here that these amino acids reciprocally regulate their release through the activation of their neuronal transporters located, as “heterotransporters”, on nerve terminals. (ii) In particular, Gly regulates the release of Glu and GABA through the activation of GlyT1 and/or GlyT2 heterotransporters and contributes to the regulation of the excitatory/inhibitory balance in different CNS areas. (iii) The transporter-mediated reciprocal regulation of release between Gly and Glu may be relevant considering the role of the two amino acids as NMDAR co-agonists. (iv) The transporter-mediated interactions among amino acid NTs occur through multiple complex, internal mechanisms that are not yet completely clarified, which lead to NT release by mechanisms different from classical exocytosis. (v) It seems increasingly clear that these phenomena can be, although not always, functional markers of cotransmission, including the possible Gly–Glu cotransmission suggested in certain CNS regions. (vi) Especially with regard to Gly–Glu, these carrier-mediated interactions can have some pathophysiological relevance in CNS pathologies in which the excitation/inhibition imbalance or NMDAR dysregulation plays a role; accordingly, some pharmacological implications may also exist.

Different compounds interacting with glycinergic, GABAergic, and glutamatergic neurotransmission and related targets (including transmitter receptors and transporters) have been receiving attention in view of the development of novel drugs. For example, pharmacological agents able to interact with GlyR isoforms, including GlyRα1 and GlyRα3 receptors, are potentially interesting for the treatment of pain (see, for instance [2,95,96]); important GlyR-related dysfunctions are involved in neurological disorders like startle disease [125,126]; GlyRs also represent possible targets for the treatment of alcohol-use disorders [127,128]. With their strong relation to the co-agonist Gly, glutamatergic NMDARs are involved in a myriad of critical neurophysiological and pathological processes, besides other ionotropic and metabotropic Glu receptors, with multiple pharmacological implications that include clinically available drugs as well as potential novel therapeutic agents [19,129,130]. GABA receptors also are highly relevant as pharmacological targets. Ionotropic GABA_A_ receptors exhibit an extremely rich pharmacology, which includes widely known therapeutic drugs such as benzodiazepine anxiolytics, antiepileptic drugs, and general anesthetics, but also more novel drugs against serious depressive states and other neuropsychiatric disorders (see [131,132]). GABA_A_ receptors, furthermore, continue to represent targets for potential novel drugs with improved features [131]. Returning to glycinergic neurotransmission, the pharmacological inhibition of GlyT1 and/or GlyT2 and the modulation of Gly receptors are currently being studied as potential therapeutic strategies against serious CNS pathologies that include pain, schizophrenia, epilepsy, depression, alcohol abuse, and neurodegenerative disorders, and some ongoing studies also include novel clinical trials [19,20,21,46,63,89,92,101,103,107,110,112,127,133,134,135,136,137,138]. In spite of the extensive and promising ongoing studies dealing with Gly receptors and Gly transporters, to date, no clinically exploitable drugs originating from these studies are yet available. It is hoped that a better understanding of the neurotransmission mediated by Gly and other amino acid NTs and the multiple functional interactions among them, particularly in Gly–Glu crosstalk, will offer some contributions.

## 9. Future Directions

Studies could be extended by exploiting the availability of genetically modified mice. As an example, mice lacking specific NT transporters in particular neuronal populations have been developed, including mice with the conditional specific deletion of GlyT1 in forebrain neurons that exhibited antipsychotic and pro-cognitive features [139,140]. More generally, studies with genetically modified animals could offer very useful information; moreover, studies could be extended to unexplored CNS areas of different animal models of CNS pathologies.

Another aspect that deserves attention relates to the so-called “metamodulation” of neurotransmission, a concept that implies that heterogeneous receptors interact with each other and/or with other structures colocalized at the same neuronal site (for instance, a nerve terminal) to control neuronal functions including NT release [141,142,143,144]. Among the many receptors involved, NMDARs are of particular interest [145,146]. Metamodulators of receptors can also include NT transporters. Functional interactions between GlyT1 transporters and NMDARs have been the object of a very recent review [19]. Transporter-mediated modulation of NT release can be interconnected with receptor-mediated mechanisms, inasmuch as NT transporters can closely interact with a colocalized receptor. With regard to Gly transporters, Musante et al. [45] found that the electrogenic GlyT1 transporter exerts a permissive role on the colocalized presynaptic NMDA autoreceptors, thus acting as an NMDAR metamodulator [45,145]. Alterations in the mechanisms of metamodulation seem increasingly relevant to CNS pathologies, as well as to the search for novel therapeutic interventions [145,146]. It will, therefore, be interesting to explore the involvement of NT transporters (including amino acid NT “heterotransporters”) in several possible mechanisms of the metamodulation of neurotransmission.

## Data Availability

No new data were created in the present research.
